# Durability analysis of metakaolin recycled concrete under sulphate dry and wet cycle

**DOI:** 10.1038/s41598-024-66803-6

**Published:** 2024-07-16

**Authors:** Chuheng Zhong, Dongping Wang, Lijuan Zhang, Weiqi Mao, Sijia Xing, Jinhui Chen, Yuan Xiao

**Affiliations:** 1 School of Civil Engineering, Architecture and Environment, Hubei University of Technology, Wuhan, 430068 China; 2 Key Laboratory of Intelligent Health Perception and Ecological Restoration of River and Lake, Ministry of Education, Hubei University of Technology, Wuhan, 430068 China; 3 China Railway Major Bridge Engineering Group Co., Ltd., Wuhan, 430050 China; 4 CBMI Construction Co., Ltd., Beijing, 100176 China; 5 Dongying Urban Construction and Development Group Co., Ltd, Dongying, 257092 China

**Keywords:** Metakaolin, Recycled fine aggregate concrete, Sulfate attack, Dry and wet cycle, Engineering, Materials science

## Abstract

This study aims to enhance the durability, cost-effectiveness, and sustainability of recycled fine aggregate concrete (RFAC) subjected to the combined effects of wet-dry cycles and sulfate erosion. Dry–wet cycle tests were conducted in RFAC with different admixtures of biotite metakaolin (MK) and 15% fly ash (FA) mix (M) under 5% sulfate erosion environment. The effect of 0%, 30%, 60% and 90% recycled fine aggregate (RFA) replacement of natural fine aggregate on mass loss, cubic compressive strength, relative dynamic modulus test of RFAC, damage modeling and prediction of damage life of concrete were investigated. The results showed that the concrete cubic compressive strength and relative dynamic modulus were optimal for recycled concrete at 15% MK biotite dosing and 60% RFA substitution, and its maximum service life was accurately predicted to be about 578 cycles under 5% sulfate dry–wet cycling using Weibull function model. This study is pioneering in addressing the durability of RFAC under sulfate attack combined with wet-dry cycling, employing a novel approach of incorporating MK and FA into RFAC. The findings highlight the practical application potential for using MK and FA in RFAC to produce durable and sustainable construction materials, particularly in sulfate-exposed environments. This research addresses a critical challenge in the construction industry, providing valuable insights for developing more durable and eco-friendly construction materials and contributing to long-term sustainability goals.

## Introduction

In today's world, sustainable development and environmental protection have become global priorities^1^. Concrete has played a significant role in construction and infrastructure development over the past few decades. However, the production process of traditional concrete has had a significant impact on the environment, including high energy consumption and carbon emissions. To address this issue, recycled concrete has emerged. Recycled concrete has lower basic mechanical properties, durability, and impermeability compared to concrete^2,3^. When evaluating the quality of concrete structures, durability is a key indicator^4,5^, with resistance to sulfate attack being an important aspect of concrete durability. In a complex process of sulfate attack, concrete exhibits two main forms of erosion: physical erosion and chemical erosion. Physical erosion is mainly reflected in the transformation of anhydrous sodium sulfate crystals to sodium sulfate decahydrate crystals, leading to an increase in crystal volume^6^. Chemical erosion from sulfate attack includes forms such as ettringite, gypsum, and calcium silicate expansion^7^, causing serious damage to the interior and exterior of concrete^8,9^.

A large number of scholars have conducted research on how to improve the sulfate resistance of recycled concrete. Boudali et al.^10^ conducted comparative experiments, and the results showed that compared to ordinary concrete, recycled concrete exhibits higher resistance to sulfate attack, indicating significant differences between the two. Xiao et al.^11^ studied the effect of the replacement rate of recycled aggregate on the sulfate resistance of recycled concrete. The results showed that the replacement rate of recycled aggregate has a minor effect on the sulfate resistance of concrete, highlighting significant differences. However, the addition of mineral admixtures has a significant impact on sulfate resistance. Ali^12^ studied the durability test of concrete by selecting a 10% admixture of metakaolin to replace part of the cement in the preparation process of concrete and selecting a 5% sodium sulfate solution as the erosion medium, the test results showed that the extension of the immersion time of the concrete in sodium sulfate solution, the admixture of metakaolin significantly enhanced the durability of the concrete.

Nabil prepared 0%, 5%, 10% and 15% metakaolin doped concrete and immersed it in 5% sodium sulfate solution and saturated lime water, and the expansion and residual strength of the concrete were determined at different ages. The test results showed that the sulfate expansion of concrete doped with metakaolin was significantly smaller than that of the baseline group concrete and stabilized with the increase of age, while the residual strength of concrete was also significantly higher than that of the baseline group at all ages^13^. Wang compared the ability of catching ash and metakaolin to resist sulfate corrosion of the mixing stop under alternating wet and dry and different test temperature environments, and the study showed that the volcanic ash effect produced by bracketed ash and metakaolin king reduces the alkalinity within the cement stone material while making the microstructure more dense, and both of them significantly improve the resistance of the mixing stop to sulfate corrosion, and the loss of strength in the dry and wet alternating environment of the miserably metakaolin stop test group was lower than that of the Silica fume test group^14^. Ding replaced cement with MK at rates of 0%, 5%, 10%, 15%, and 20%, and used 50% and 100% recycled coarse aggregate to replace natural aggregate. They found that the optimal sulfate resistance was achieved with 15% MK replacement, while the effect of higher levels of recycled aggregate replacement was poor^15^. Al-Dulaijan^16^, Tikalsky, and Carrasquillo^17^ studied the effect of fly ash on sulfate resistance. The study showed that mortar with 20% fly ash replacing an equal amount of cement has better sulfate resistance than ordinary cement mortar, with the optimal fly ash content being 25%. Rattapon Somna found that using fly ash instead of cement reduces the sulfate expansion of recycled aggregate concrete below that of ordinary concrete, thereby improving sulfate resistance. It can be seen that MK and FA can enhance the sulfate resistance of recycled concrete^18^.

In this study, metakaolin and fly ash were used as composite auxiliary cementitious materials with recycled concrete fine aggregate to prepare recycled concrete. Concrete with 0% of metakaolin and recycled fine aggregate mixing was used as the reference group of specimens to compare and analyze the cubic compressive strength, relative dynamic modulus, mass loss, and the influence of different types of concrete under the double influence of sulfate dry and wet cycles, and the damage model was established by using the experimental data, and the microscopic morphology was observed by combining with SEM. Weibull theory was also used to predict the damage life of concrete and to analyze the effect of biased kaolin admixture and recycled fine aggregate replacement rate on the life of concrete. Based on the recycled concrete and durability characteristics, the optimal mixing amount of metakaolin in recycled fine aggregate concrete under different recycled fine aggregate substitution rates is summarized. In order to improve the research theory on the sulfate erosion resistance of this type of recycled fine aggregate concrete and expand its application in the field of sulfate wet and dry cycles. Recycled fine aggregate concrete hold the potential for diminishing environmental impact, streamlining resource utilization, and advocating for circular economy principles within the construction industry.

## Material and sample preparation

### Raw material

**Figure 1:**
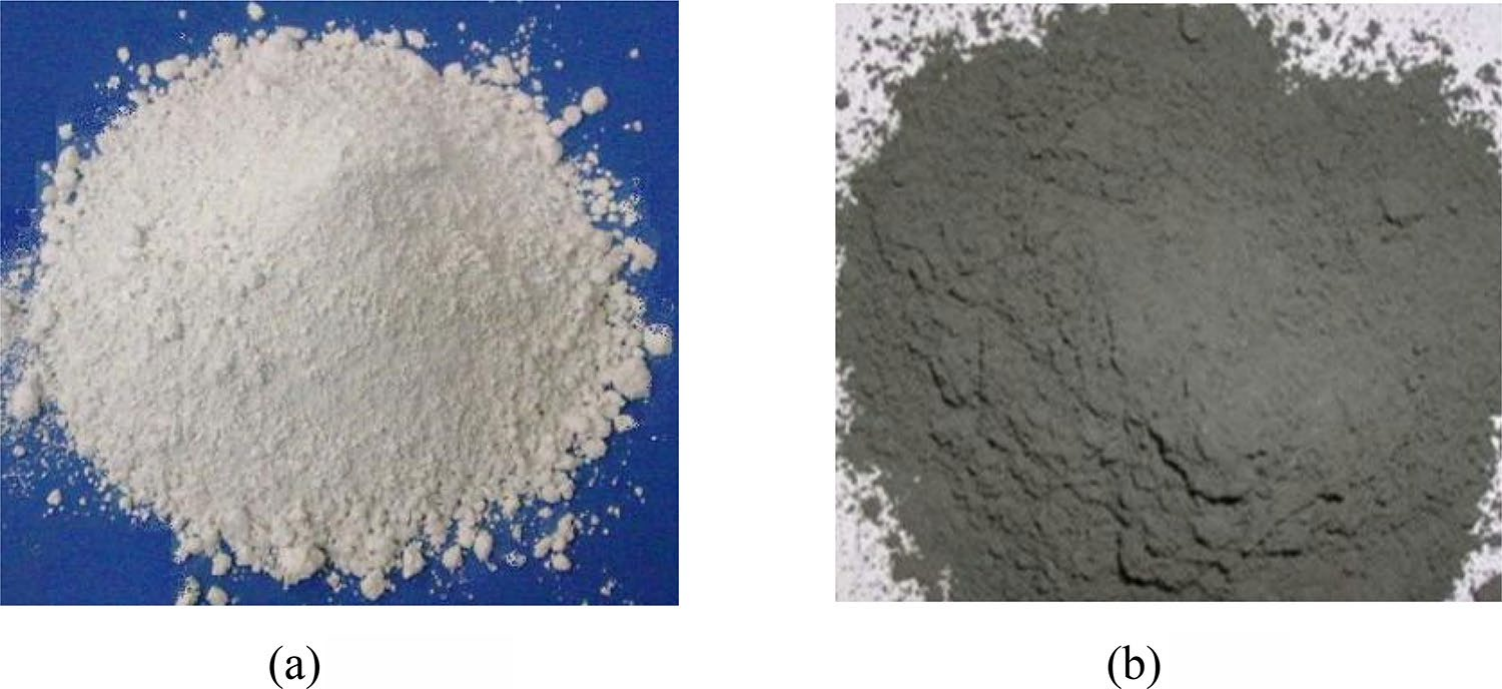
MK and FA apparent sample maps. (**a**) MK, (**b**) FA.

**Figure 2:**
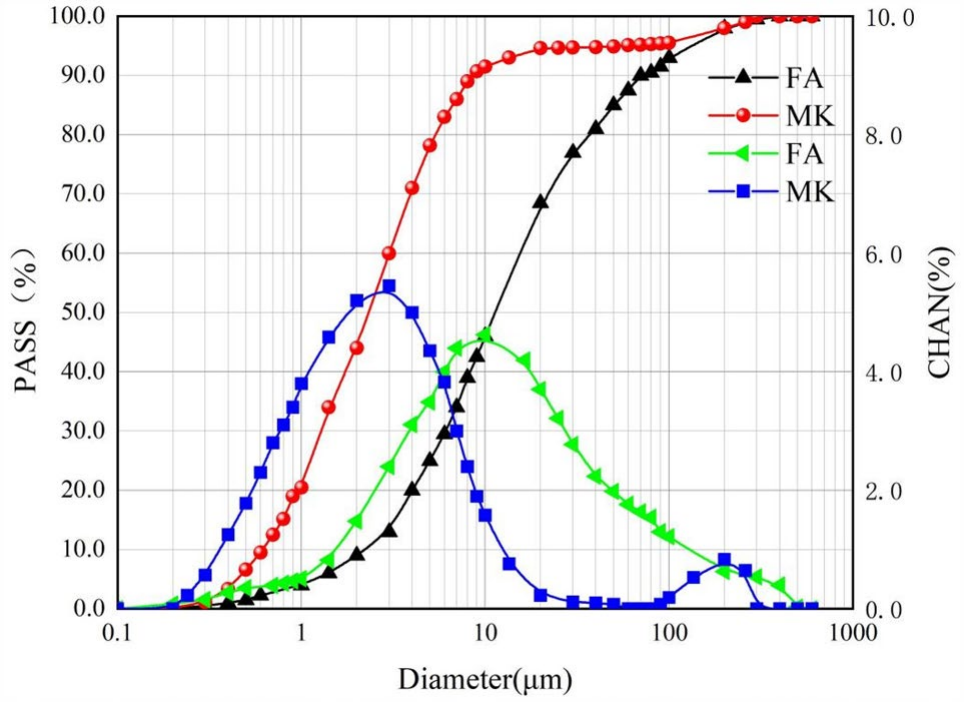
Particle size distribution of MK and FA.

**Figure 3:**
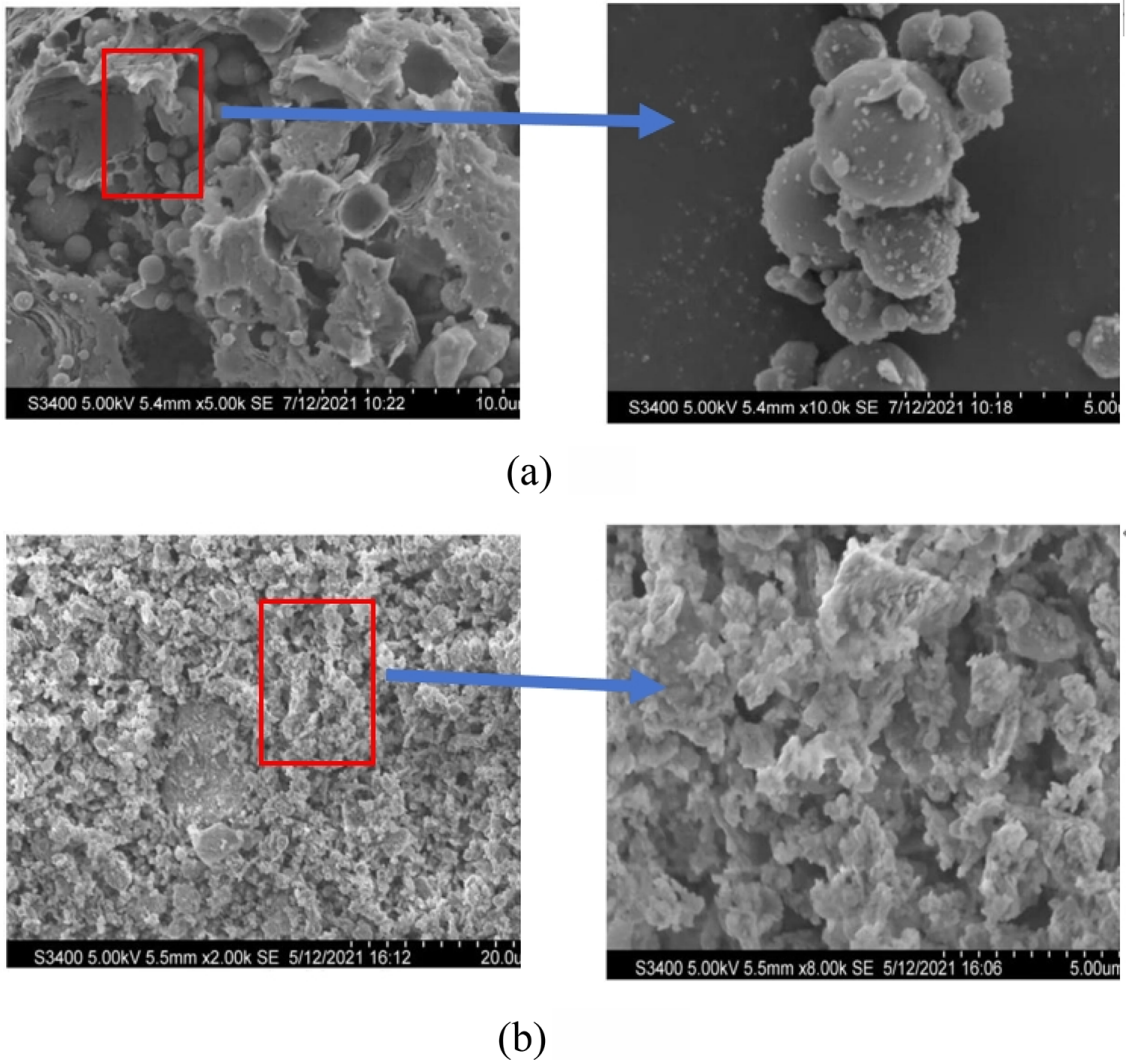
SEM of (**a**) FA and (**b**) MK.

The cementitious materials used are P.O.42.5 grade ordinary silicate cement (OPC), metakaolin (MK), and Class F fly ash (FA). OPC is purchased from Wuhan Leibi Tengda Building Material Co., Ltd. in China, and MK and FA are selected from Wuhan Huashen Zhineng Green Building Material Co., Ltd. in China, with the high activity metakaolin and Class F fly ash, as shown in Fig. 1. MK and FA apparent sample maps. Please refer to Table 1 for detailed chemical composition and physical properties. The particle size gradation and microscopic morphology of both are shown in Figs. 2 and 3.

**Table 1:** Some physical, chemical and mechanical properties of using materials.

Chemical composition (%)				Physical properties of cement		
	OPC	FA	MK	Initial setting time (min)		192
SiO_2_	20.15	47.7	46.82	Final setting time (min)		257
Al_2_O_3_	5.02	37.53	50.46	Compressive strength (MPa)		49. 8
Fe_2_O_3_	2.83	4.55	0.44	Fineness (%)	OPC	0. 32
CaO	60.13	3.70	0.10		FA	10. 6
MgO	3.52	0.94	0.13		MK	0. 0
K_2_O	0.68	1.62	0.11	Pozzolanic activity index (%)	FA	72
Na_2_O	0.20	0.60	0.26		MK	115
TiO_2_	–	1.40	1.16	Heat loss	OPC	3. 13
P_2_O_5_	–	0.36	0.34		FA	4. 32
SO_3_	3.47	0.91	–

**Figure 4:**
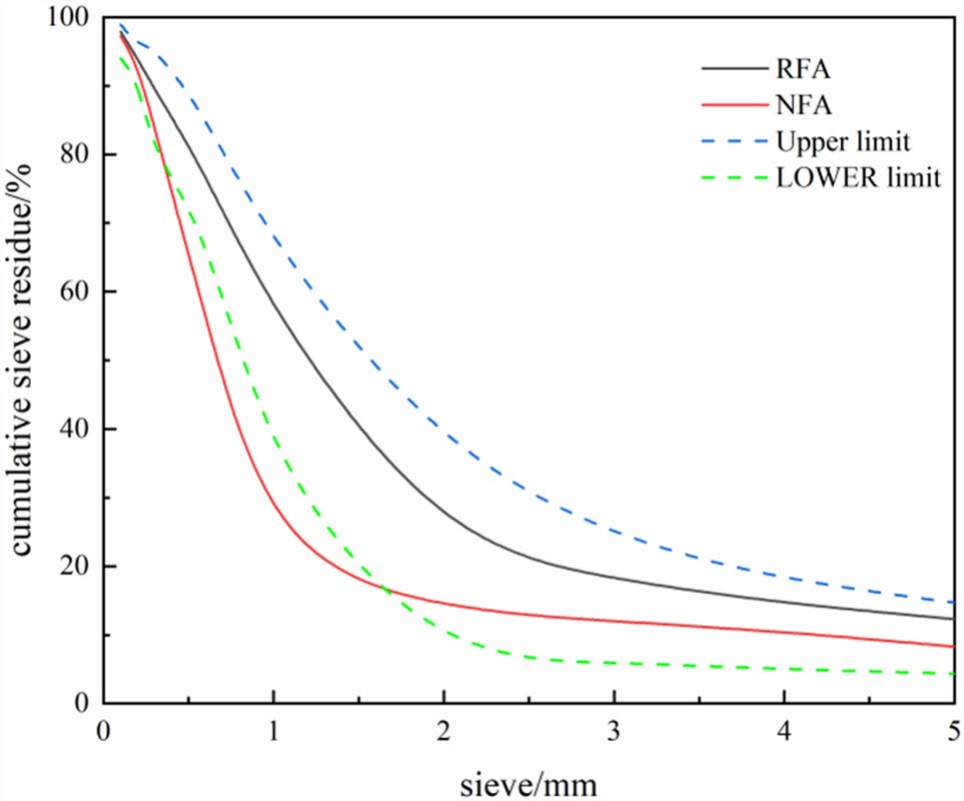
Aggregate gradation.

The fine aggregates used are natural river sand (NFA) and recycled concrete fine aggregate (RFA), with the gradation curve shown in the Fig. 4. Aggregate gradation. NFA, the natural aggregate, is obtained through natural extraction and processing of river sand, purchased from Leibei Tengda Building Materials Co., Ltd. in Wuhan. RFA is derived from laboratory discarded concrete test specimens with an initial strength of C35. It is crushed, sieved, soaked, washed with clean water, and air-dried to obtain a particle size range of 0.15–4.75 mm. The properties of the aggregates are presented in Table 2. The coarse aggregate (CA) used is 5–20 mm crushed limestone with a continuous gradation of particle sizes.

**Table 2:** Aggregate basic performance index.

Aggregate type	Apparent density	Bulk density	Crushing index	Water absorption
5–20 mm CA	2695	1340	3.98	0.43
0.15–4.75 mm RFA	2450	1230	–	5
0.15–4.75 mm NFA	2650	1445	–	1.82

Polyvinyl alcohol fibers (PVA) with a length of 18 mm were selected from Shanghai Chemical Building Materials Additives Company in Shanghai, China. In order to avoid the agglomeration phenomenon when mixing PVA with other mixes to affect the test results, it will be refined by manual treatment. The specific physical properties are listed in Table 3.

**Table 3:** The physical properties of PVA fiber.

Length/mm	Diameter/µm	Density/g. cm^3^	Elastic modulus/GPa	Elongation/%
18	18	1.29	37.5	7

In this experiment, 0.4% (by mass of the total cementitious materials) of a high-efficiency water reducer was added. The polycarboxylic acid-based high-efficiency water reducer powder (SP) produced by Jiangsu Suqian Home Decoration Building Materials Co., Ltd. was used. The excellent water retention of the water reducer can moderately reduce the water content of recycled concrete, reduce the viscosity of the mixture, improve the flowability of recycled concrete, and achieve a water reduction rate of 20%. Analytical grade anhydrous sodium sulfate (AR Na_2_SO_4_) was used, and a relative molecular mass of 142.04. The mixing water was local tap water from Wuhan.

### Mix design and procedure

In this study, a total of 13 mixtures were prepared using C40 ordinary concrete as the reference. Under the conditions of a water-to-cementitious materials ratio of 0.42 and a total binder content of 410 kg/m^3^, the mixtures were formulated. The mixtures prepared using OPC, MK, and FA are referred to as "M." The ternary mixtures, where MK replaced 5%, 10%, and 15% of OPC, were named "M5", "M10" and "M15", respectively. The replacement proportions of RFA for NFA were 0%, 30%, 60%, and 90%, and they were named "RFA0", "RFA30", "RFA60" and "RFA90," respectively. Among all the combinations, FA and PVA are dosed into the compound. The mixing ratios of the 13 mixtures are listed in Table 4.

**Table 4:** The M/RFAC mix ratio design (kg/m^3^).

Mix	OPC	MK	FA	CA	NFA	RFA	Water	SP	PVA
M0RFA0	410	0	0	1214.2	653.8	0	172.2	1.3	1.29
M5RFA0	328	20.5	61.5	1214.2	653.8	0	172.2	1.64	1.29
M5RFA30	328	20.5	61.5	1214.2	457.7	196.1	172.2	1.64	1.29
M5RFA60	328	20.5	61.5	1214.2	261.5	392.3	172.2	1.64	1.29
M5RFA90	328	20.5	61.5	1214.2	65.4	588.4	172.2	1.64	1.29
M10RFA0	307.5	41	61.5	1214.2	653.8	0	172.2	1.64	1.29
M10RFA30	307.5	41	61.5	1214.2	457.7	196.1	172.2	1.64	1.29
M10RFA60	307.5	41	61.5	1214.2	261.5	392.3	172.2	1.64	1.29
M10RFA90	307.5	41	61.5	1214.2	65.4	588.4	172.2	1.64	1.29
M15RFA0	287	61.5	61.5	1214.2	653.8	0	172.2	1.64	1.29
M15RFA30	287	61.5	61.5	1214.2	457.7	196.1	172.2	1.64	1.29
M15RFA60	287	61.5	61.5	1214.2	261.5	392.3	172.2	1.64	1.29
M15RFA90	287	61.5	61.5	1214.2	65.4	588.4	172.2	1.64	1.29

There is a significant difference between the water absorption of RFA and NFA, in order to avoid the mortar attached to the surface of RFA during the mixing process of recycled concrete to absorb part of the mixing water, which leads to the reduction of the water-cement ratio of RFAC. Therefore, in the test, the method of "RFA secondary grinding + RFA pre-wetting + batch mixing" was used to prepare recycled concrete.

**Figure 5:**
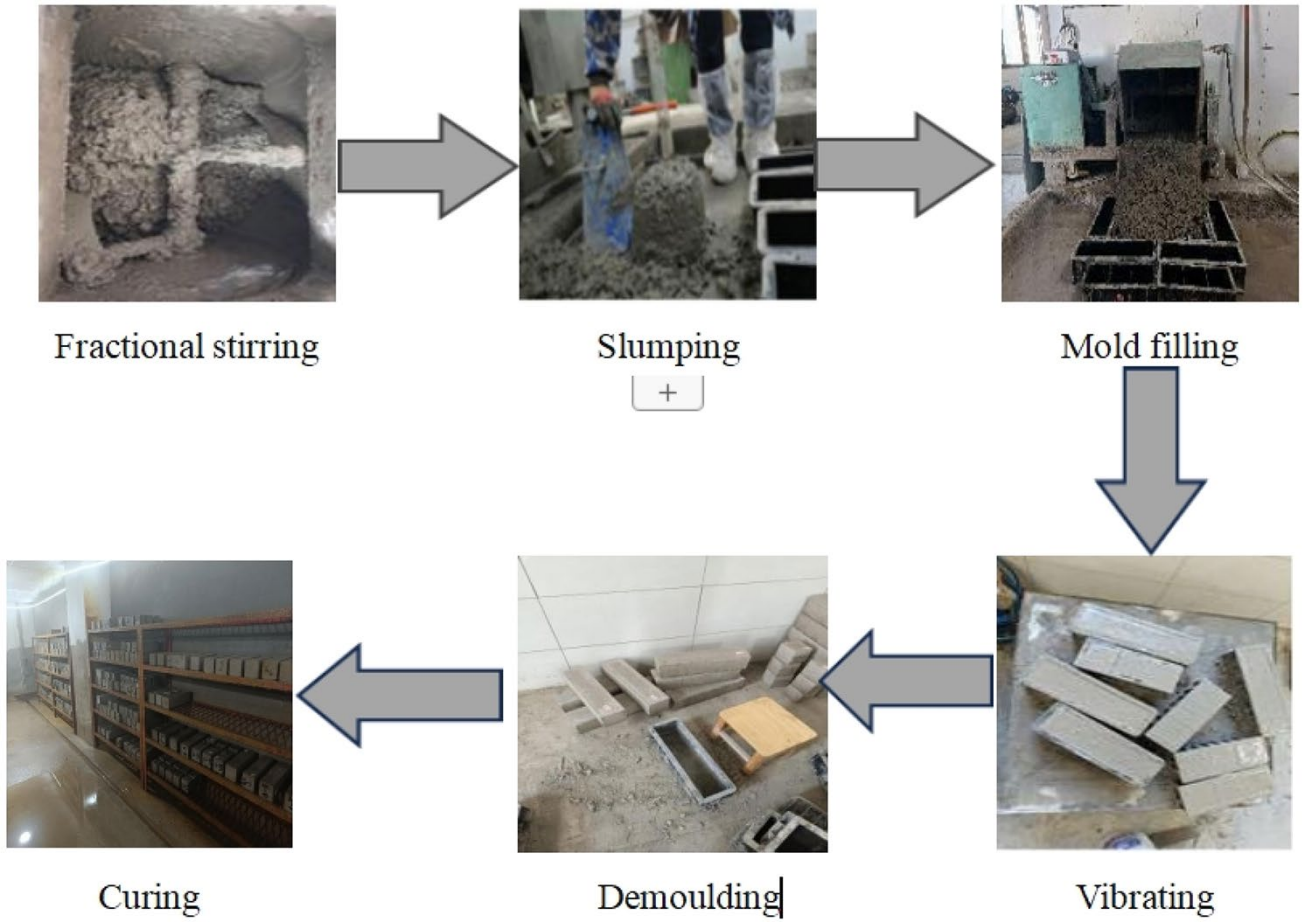
Concrete preparation process.

Concrete test specimens for recycled concrete were prepared according to the mix proportions in Table 4. The specific process is shown in Fig. 5. Concrete preparation process. Test preparation (100 mm × 100 mm × 100 mm) of cubic test blocks a total of 312, (100 mm × 100 mm × 400 mm) of rectangular test blocks a total of 39, 13 groups of test blocks each group of 24 cubic test blocks and 3 rectangular test blocks. The production and curing of concrete specimens followed the requirements of the "Standard Test Methods for Mechanical Properties of Concrete"(GB/T50081-2019)^19^. After curing, the specimens were removed, surface moisture was wiped dry, and they were placed in a drying oven at (80 ± 5) °C for 48 h for storage. During the experiment, cubic specimens and rectangular specimens were subjected to dry–wet sulfate erosion tests simultaneously. The cubic specimens were used for post-cycling compressive strength tests, while the rectangular specimens were used to measure the mass loss and dynamic modulus after cycling.

## Tests and test methods

**Figure 6:**
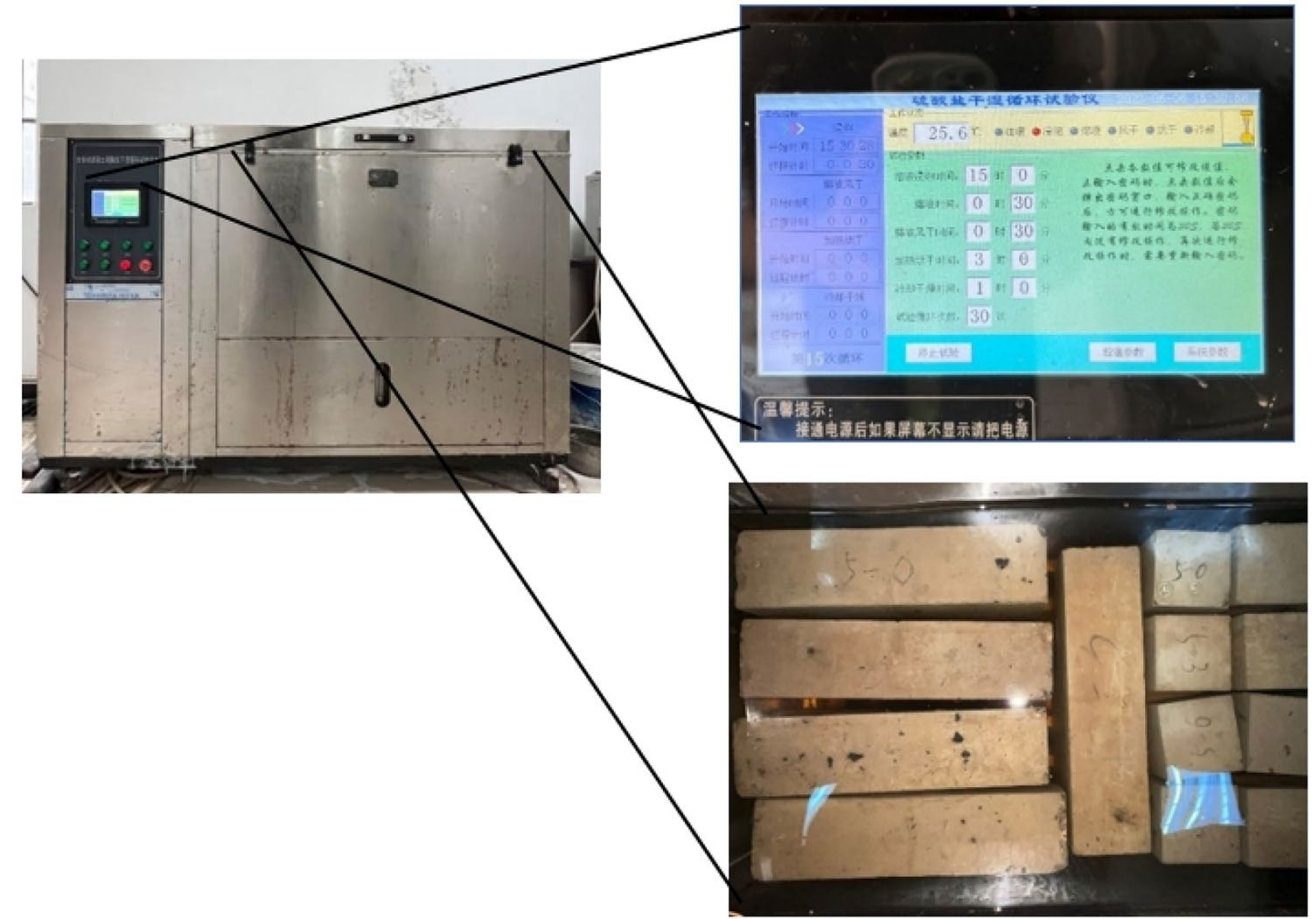
M/RFAC sulfate erosion coupled dry–wet cycle test.

**Figure 7:**
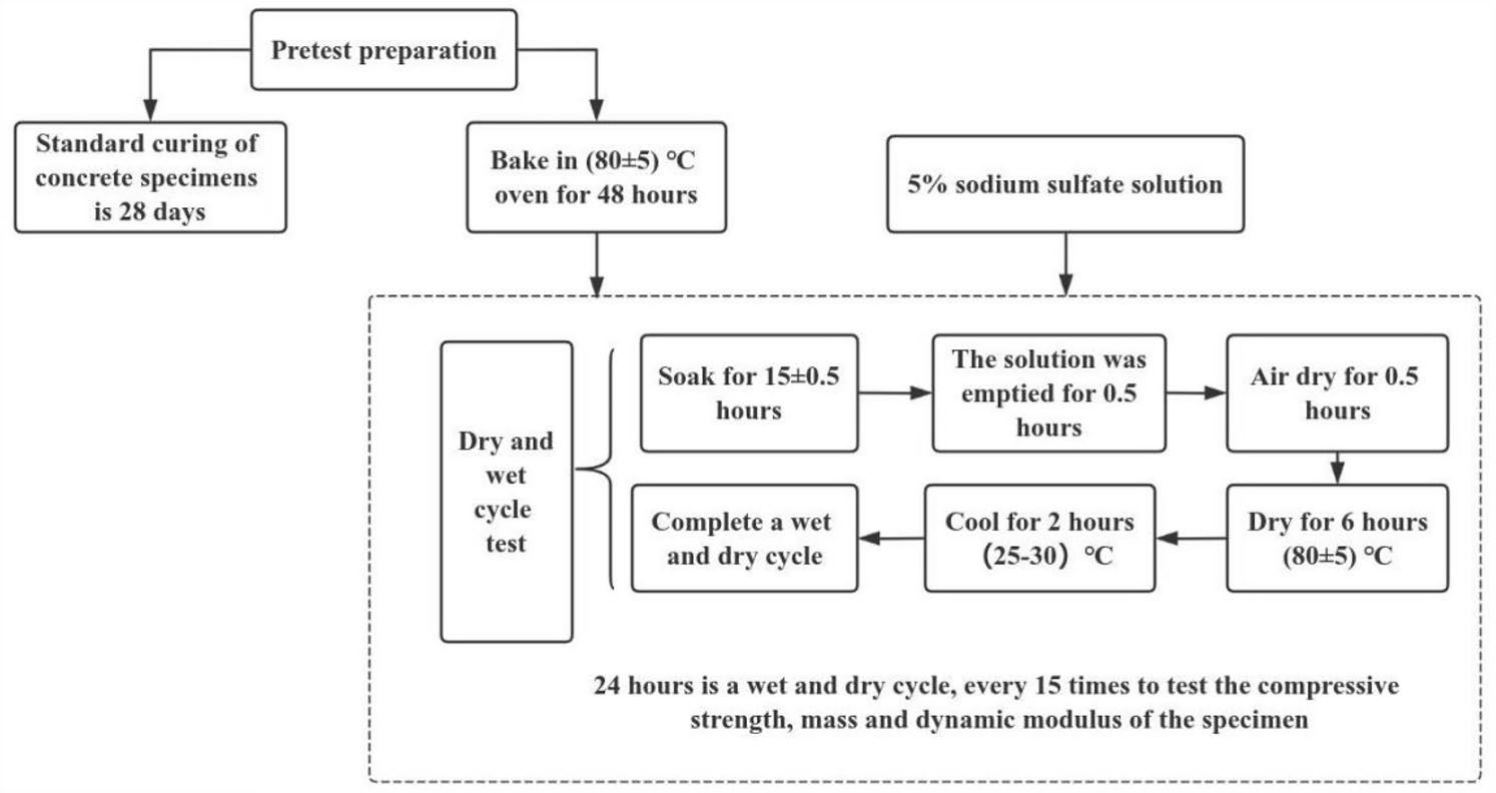
Concrete dry–wet cycle test process.

LSY-18 type sulfate wet-dry cycle equipment was selected for indoor rapid wet-dry cycle tests. The testing equipment is shown in Fig. 6, and the specific steps are illustrated in the Fig. 7. During each specified test cycle, the pH value of the solution in the chamber was monitored to ensure it remained around 7. If not, the solution was reconfigured^20^. After 0, 15, 30, 45, 60, 75, 90, 105, 120 wet and dry cycles, test the dynamic elastic modulus and mass loss of the specimen with DT-20 dynamic elastic modulus tester on the specimen with the size of 100 mm × 100 mm × 400 mm, three for each group, and test the dynamic elastic modulus and mass loss of the specimen with the size of 100 × 100 × 100 mm using DYE-2000S microcomputer servo pressure testing machine, and test the cubic compressive strength of the specimen with the size of 100 × 100 × 100, three for each group. For 100 × 100 × 100 mm specimens of the cubic compressive strength, three per group. The specimens tested for 120 times were retained for SEM microanalysis using a microscope.

### Mass loss

Measure the mass change of recycled concrete using an electronic scale with a precision of 0.001 kg. Record the mass of concrete with different mix ratios for each wet and dry cycle. Calculate the average of the three test results for each set of specimens as the measured value.

### Loss of compressive strength

**Figure 8:**
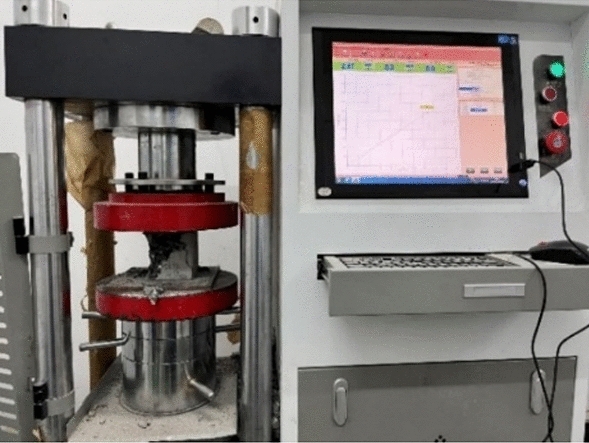
Concrete compressive strength test equipment.

Use the DYE-2000S microcomputer servo pressure testing machine to test the cube compressive strength of the specimens. The testing instrument is shown in Fig. 8.

The rate of loss of compressive strength of recycled RFA is calculated as in Eq. (1) .

1 $${f}_{x}=\frac{{f}_{i}-{f}_{0}}{{f}_{0}}\times 100\%,$$

where *f*_*x*_ is the compressive strength loss rate after i dry–wet cycle, %;* f*_*x*_ is the compressive strength after i dry–wet cycle, MPa; *f*_*0*_ is the initial compressive strength without cycling, MPa.

### Relative dynamic modulus value

**Figure 9:**
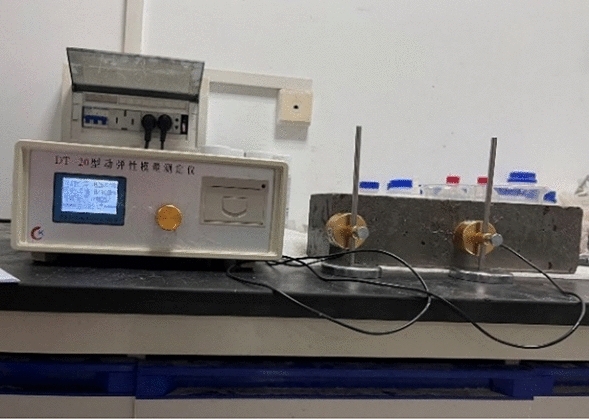
M/RFA dynamic elastic modulus measurement test.

The test apparatus is shown in Fig. 9.

The *Ei* of RFAC is calculated in the following Eq. (2). In this test, the average test results of three specimens were used as the final determination.

2 $${E}_{i}=\frac{{f}_{i}^{2}}{{f}_{0}^{2}}\times 100\%,$$

where *E*_*i*_ is the relative dynamic modulus of recycled concrete specimen after *i* dry–wet cycle, %; Is the natural vibration frequency of the concrete specimen before the dry–wet cycle, Hz; Is the natural vibration frequency after *i* dry–wet cycle, Hz.

### Apparent damage and microscopic analysis

At the end of each dry–wet cycle, observe the appearance changes of each group of specimens, take photos, and record them. Compare and analyze the different appearances of recycled concrete erosion with different replacement rates of recycled fine aggregate and dosages of MK, and preliminarily describe the degree of damage. After 120 cycles, scan the specimens with SEM electron microscopy. The microscopic images can visually show the changes in cracks and voids inside the recycled concrete after dry–wet cycles, as well as the influence of high-activity mineral admixtures on recycled concrete. This provides a visual explanation for the macroscopic index decline of recycled concrete after sulfate dry–wet cycles.

## Results and discussion

### Compressive strength analysis

#### Influence of different dosage of MK on compressive strength of M/RFAC

**Figure 10:**
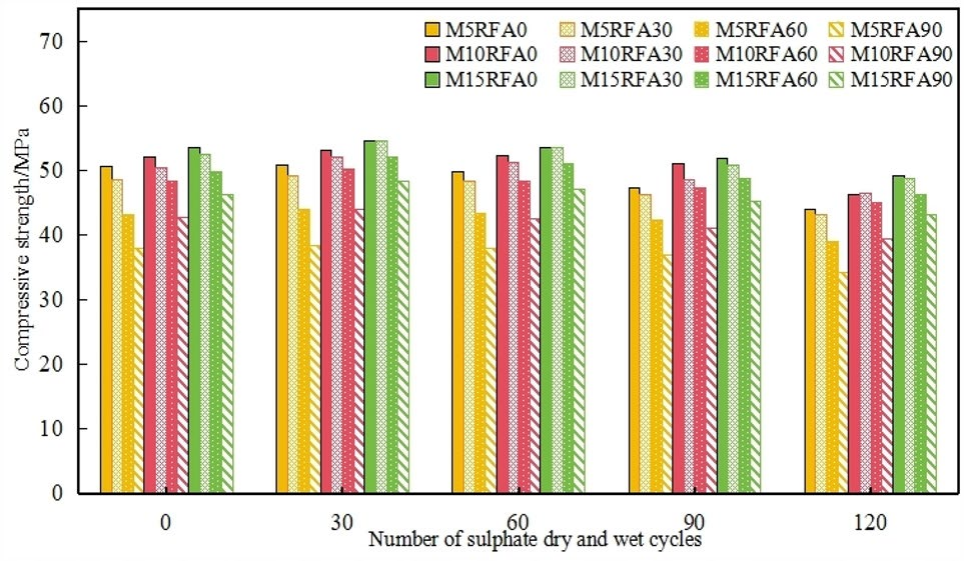
M/RFAC Compressive strength of cube.

With the increase in erosion cycles, in Fig. 10, The compressive strength of each group of M/RFAC showed a slight increase in the first part of the cycle, a slow decrease in the middle part of the cycle, and an increase in the decrease of compressive strength in the late part of the cycle. The mechanism behind this trend is as follows: in the early stage of sulfate cycles, sulfate ions infiltrate and react to form C–S–H, filling the pores inside the concrete, which densifies the internal structure and thus improves the compressive strength. In the later stages of the cycles, temperature stress, expansion stress, and salt crystallization stress increase the internal pore pressure of the concrete. This leads to the appearance of microcracks, which continue to expand with the increase in cycle number, accelerating the infiltration of sulfate ions. Further erosion reactions destroy the structure of the cement stone, and the precipitation of cementitious material solution causes the compressive strength to continuously decrease. Among them, the compressive strength of concrete M5RFA0, M10RFA0, and M15RFA0 reaches its peak after 30 erosion cycles, at 50.9 MPa, 53.0 MPa, and 54.5 MPa respectively. At the same number of erosion cycles, the compressive strength of concrete is arranged in the following order:M15 > M10 > M5, indicating that the addition of MK increases the compressive strength of concrete under the dual effects of sulfate erosion and dry–wet cycles. This is consistent with the findings of Ali et al. and Rakesh et al.^20,21^. After the addition of MK to concrete, it reacts with calcium hydroxide produced by the hydration process of cement to form an additional gelling C–S–H gel, which changes the microstructure of concrete, as shown in Fig. 17, and the gelling material formed makes the concrete more tightly connected, which improves the strength. Especially in the early stages^21^. In addition, the fine particles of MK penetrate into the spaces between the cement particles, thereby densifying ITZ and the overall microstructure of the concrete system, which helps to reduce the sulfate erosion of the cement matrix by thinning the pores in the cement.

**Figure 11:**
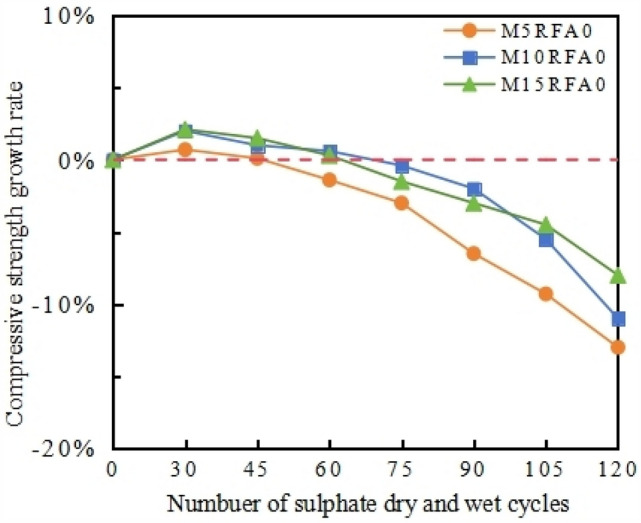
Relationship between the growth rate of compressive strength of Group RFA0 and the number of cycles.

To further analyze the effect of MK content on the compressive strength of concrete under cyclic erosion, a line graph depicting the relationship between the compressive strength growth rate of the RFA0 group concrete and the number of dry–wet cycles was plotted, as shown in Fig. 11. Compared with M10 and M0, the decline trend of M15 is gentle. Within 45 cycles, the addition of MK resulted in varying degrees of improvement in concrete strength, increasing by 0.1%, 1.0% and 1.5% respectively. Among them, with the increase in cycle number to 60 cycles, the compressive strength of M10RFA0 and M15RFA0 increased compared to the initial strength, by 0.6% and 0.3%, respectively. This is mainly due to the significant optimization of the concrete pore size distribution structure with the addition of MK and FA. As Fig. 17d. Furthermore, the mixed compound has a good pozzolanic activity, when the hydration reaction occurs, the generated C–S–H and hydrated calcium aluminate and other cementing substances increase the density of the concrete structure, that is, the pozzolanic effect: Eqs. (3), (4).

3 $$xCa(OH)_{2} + SiO_{2} + mH_{2} O \to xCaO \cdot SiO_{2} \cdot nH_{2} O,$$

4 $$xCa(OH)_{2} + Al_{2} O_{3} + mH_{2} O \to xCaO \cdot Al_{2} O_{3} \cdot nH_{2} O.$$

Meanwhile, FA and MK undergo secondary hydration reactions in the solution, consuming a large amount of hydrated calcium silicate in the cement paste. This reduces the formation of excessive expansive products in the reaction between cement paste and sulfate.

#### Influence of RFA on the compressive strength of M/RFAC

**Figure 12:**
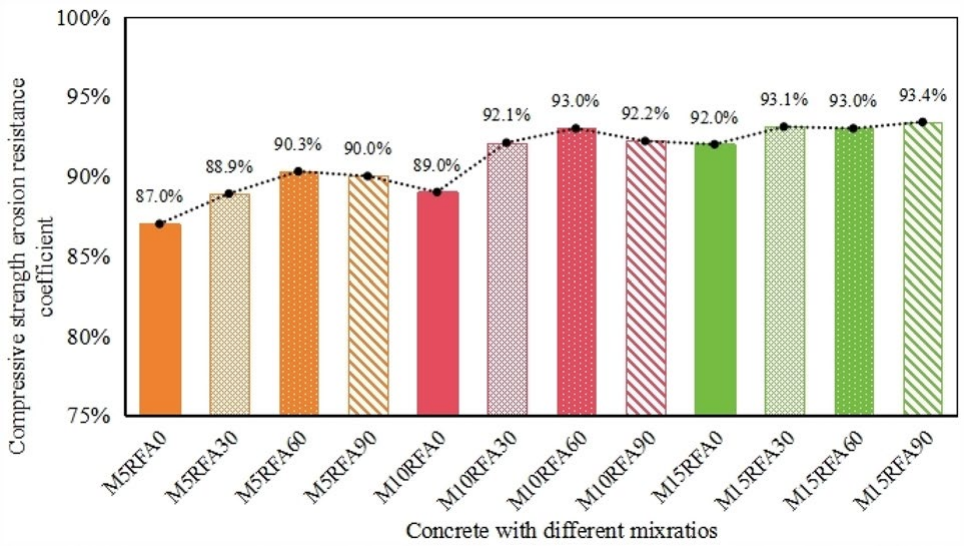
Compressive strength and corrosion resistance coefficient of RFA after 120 erosion cycles.

Figure 12 shows a clear trend in the compressive strength durability coefficient (*r*) of recycled concrete as the replacement rate of recycled aggregate increases. Initially, r tends to rise, but then it gradually decreases again, with a turning point at 30 cycles. After undergoing 120 cycles of sulfate erosion and wet-dry cycles, the compressive strength durability coefficients of specimens M5RFA30, M5RFA60, and M5RFA90 were 88.9%, 90.3%, and 90.0% respectively. This indicates that *r* increases with the increase in the replacement rate of recycled aggregate, reaching its maximum value at a 60% replacement rate. It indicates that RFA with a certain admixture exhibits higher resistance to sulfate attack compared to plain concrete, which is similar to the experimental results of Boudali et al.^10^. According to previous studies^22^, As Fig. 17c, during sulfate erosion, the hydration products of cement, such as Ca(OH)_2_ and C_3_A, react chemically with ${\text{SO}}_{{4}}^{{{2}^{ - } }}$ to form ettringite (C_6_AS_3_H_32_) and gypsum(CSH_2_) (Eqs. 5 and 6). These erosion products can fill the voids in mortar, making the original concrete denser and reducing the water absorption of recycled aggregate. However, when the replacement rate reaches 90%, the compressive strength durability coefficient of RFAC decreases. This is because excessive incorporation of recycled fine aggregate affects the hydration of cement, creating a weak layer at the interface between cement and aggregate, preventing their full integration^23^. Additionally, excessive ettringite production increases the internal solid volume, leading to significant stress from mutual compression of ettringite. As Fig. 17d. When the accumulated stress exceeds the internal tensile strength of concrete, the internal structure of recycled concrete is damaged, the bond between local aggregate and cement disappears, and the compressive strength of RFA90 decreases. The excessive addition of RFA reduced the mechanical properties of concrete, similar to the results of Ju et al.^24^. In specimens of RFAC in group M10, the compressive strength durability coefficients of M10RFA30, M10RFA60, and M10RFA90 were 92.1%, 93.0%, and 92.2% respectively. Similarly at 60% RFA replacement, the r value of RFAC is maximum, and the same result is obtained in group M15. It can be seen that the best performance of RFAC against sulfate attack and wet-dry cycling was achieved at 60% substitution rate for a certain dosage of MK.

5 $$Ca(OH)_{2} + SO_{4}^{2 - } + 2H_{2} O \to C\overline{S}H_{2} + 2OH^{ - } ,$$

6 $$C_{3} A + 3C\overline{S}H_{2} + 26H_{2} O \to C_{6} A\overline{S}_{3} H_{32} .$$

### Relative dynamic modulus analysis

**Figure 13:**
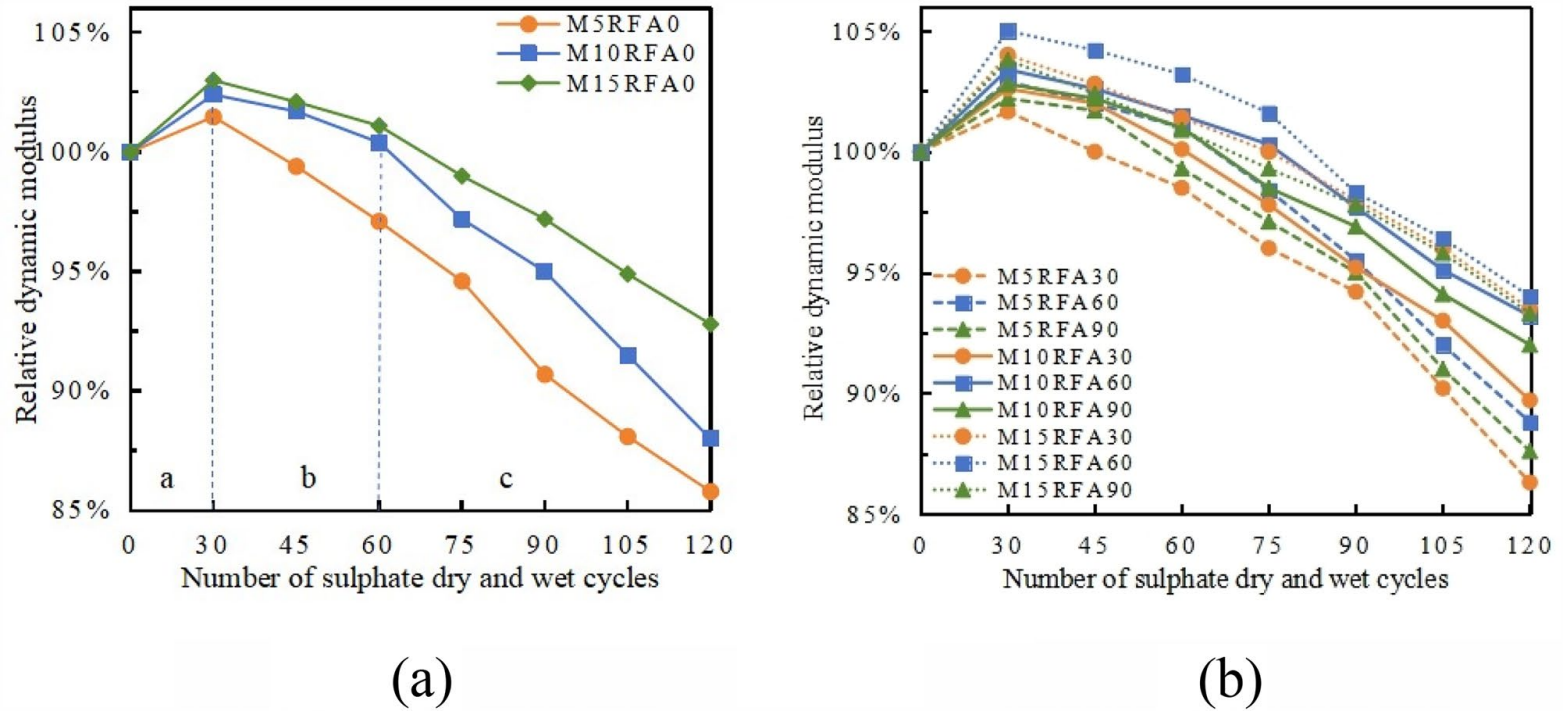
The relation between the relative dynamic modulus of RFA with the number of cycles under different MK content. (**a**) RFA0, (**b**) RFA30, RFA60, RFA90.

The relationship between the relative dynamic modulus of elasticity of concrete and recycled aggregate concrete and the replacement rate of recycled aggregate, as well as the amount of MK, is shown in Fig. 13.

From Fig. 13a, The overall trend of dynamic modulus change is that the relative dynamic modulus of M/RFAC specimens increases rapidly when the number of wet and dry cycles is less than 30 times. As the erosion continues until 30 cycles, the relative dynamic modulus of elasticity gradually decreases overall, with a slight decrease during the mid-term 60 cycles of erosion, but then decreases more rapidly in the later stage. Within 120 cycles, the relative dynamic modulus of concrete and RFAC undergoes three stages of change: a rapid increase stage a, a slow decrease stage b, and a rapid decrease stage c. The curve shows that with an increase in the amount of MK, the decrease in stages b and c is significantly reduced. In the RFA0 group, M15RFA0 shows the smallest decrease, reaching 92.8%. This indicates that MK significantly improves the resistance of concrete to sodium sulfate corrosion. Previous literature also reported similar effects of MK content on RFAC strength development^25^. This is mainly because the erosion solution provides conditions for the rehydration of concrete, promoting the volcanic ash effect of MK and FA in the concrete matrix, forming more stable compounds, slowing down the rate of sulfate ion intrusion into the concrete, improving the ductility of RAC, increasing the deformation capacity of specimens, and thereby improving the resistance of concrete to sodium sulfate corrosion^26^. Additionally, the production of dense Ca(SO_4_)_2_ fills the pores inside the concrete, increases the density of the concrete, and improves its resistance to sodium sulfate corrosion.

From Fig. 16b, it can be observed that the change in relative dynamic modulus of RFAC with MK is similar to that of concrete and plays a good reinforcing role. The order of enhancement magnitude is M15 > M10 > M5. This indicates that sulfate wet-dry cycle erosion also provides a similar reaction environment for recycled concrete, allowing the mineral admixtures and hydration products of cement in recycled concrete to undergo secondary hydration reactions, producing ettringite and calcium silicate hydrate (C–S–H) and other products, filling some of the pores in the concrete, making the concrete denser than before the cyclic test in the early stages of the cycle. This is similar to J. Haufe et al.^27^. However, with the arrival of erosion stages b and c, the hydration products in the pores continue to increase, exerting pressure on the pore walls, causing the internal expansion stress of the specimen to accumulate continuously, eventually leading to cracks inside the specimen. The expansion of internal cracks manifests as a decrease in the relative dynamic modulus of elasticity of the specimen macroscopically, affecting the mechanical properties and durability of the concrete^22,23^.

### Mass loss rate

#### Effect of RFA substitution rate on M/RFAC mass loss rate

**Figure 14:**
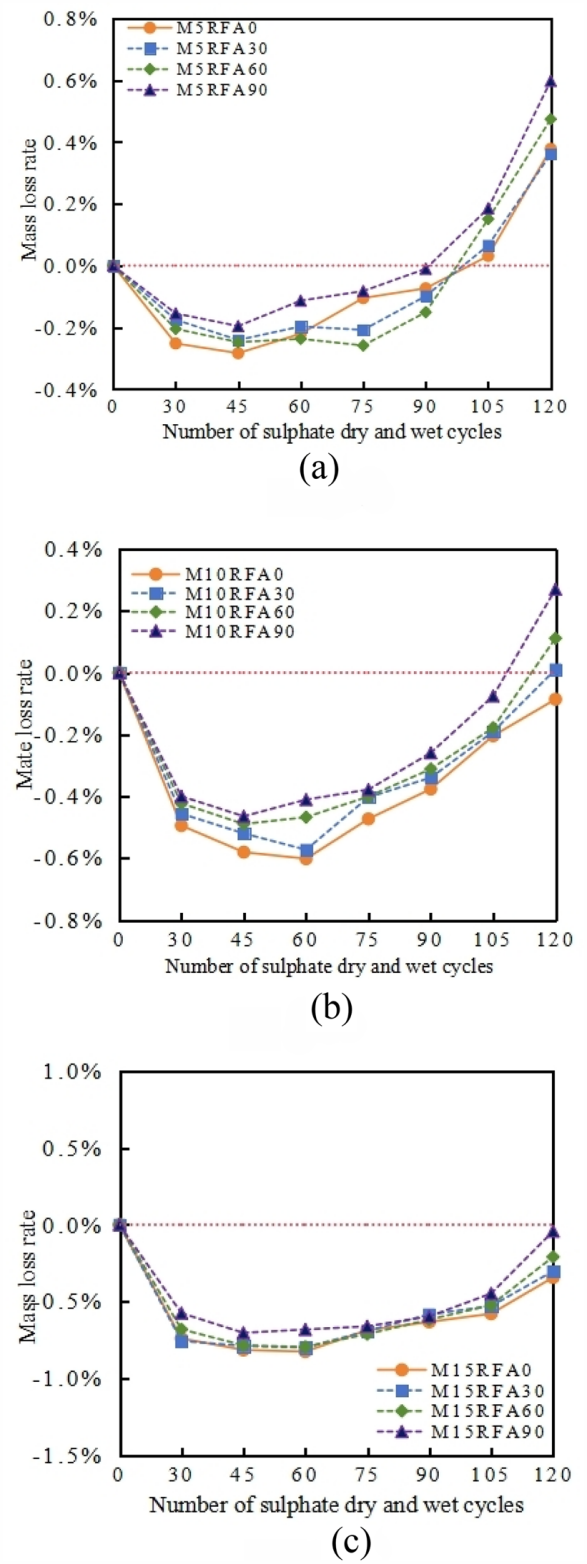
The relationship of M/RFAC mass loss rate with the number of cycles under different RFA content. (**a**) M5, (**b**) M10, (**c**) M15.

The mass loss rate of M/RFAC with MK content of 5%, 10%, and 15% after each sulfate dry–wet cycle varies with the number of cycles, as shown in Fig. 14.

After 120 cycles of dry–wet exposure in a 5% sodium sulfate solution, there is a certain difference in the mass loss rate between group RFA0 concrete and group RFA30, RFA60, and RFA90 recycled concrete. When the number of dry–wet cycles is less than 30, the differences in mass loss rates among the various M/RFAC groups are small, and the mass increases the most, reaching 0.18%, 0.20%, 0.15% for RFA30, RFA60, RFA90 respectively. As the erosion continues, the increase in mass after 45 cycles significantly slows down compared to 30 cycles, with only 0.07%, 0.04%, 0.04% for RFA30, RFA60, RFA90 respectively. However, the mass of RFAC continues to increase. The analysis shows that in the early stage of erosion, RFAC absorbs sulfate quickly due to the high water absorption of the aggregate, which leads to the rapid absorption of sulfate into the concrete and reacts with the hydration products of cement to form expansive corrosion substances such as gypsum and ettringite^28^, causing the mass of concrete to increase rapidly. As the sulfate-dry–wet cycle erosion progresses, the mass loss rates of the specimens in each group begin to rise, and the rate of mass loss accelerates. This indicates that as the erosion progresses, the erosion substances formed in the later stages of the cycle increase, causing expansion stress to increase, pore cracks to expand, and the sulfate corrosion rate to increase, manifesting as the formation of more expansive erosion substances, internal crack expansion, and mortar spalling, as shown in Fig. 16. This is reflected in a rapid decrease in mass^29^. When the number of cycles reaches 120, the mass loss rate of the specimens containing 90% recycled fine aggregate is the highest, reaching 0.6%, 0.27%, 0.04% respectively, and compared with group RFA0 concrete, it increases by 0.22%, 0.36%, 0.30% respectively. This indicates that the higher the content of RFA, the greater the mass loss. Similar results were found in literature^30^, and after 75 dry–wet cycles, the mass loss rate accelerates. The order of mass loss rate increase rate is RFA90 > RFA60 > RFA30 > RFA0. The main reason is that sulfate erosion leads to a decrease in the bonding strength of recycled concrete. Sulfate erosion can cause concrete dissolution and damage. In addition, the temperature of the dry–wet cycle test is 80 °C. In a high-temperature environment, the hydration reaction rate of cementitious materials accelerates, leading to rapid early strength development of concrete. However, excessive hydration reaction may lead to internal stress concentration and crack formation, resulting in reduced concrete strength. These two reasons together lead to the destruction of the macroscopic surface mortar of recycled concrete, resulting in mass loss, as shown in Fig. 16.

#### Effect of MK content on quality loss rate of M/RFAC

**Figure 15:**
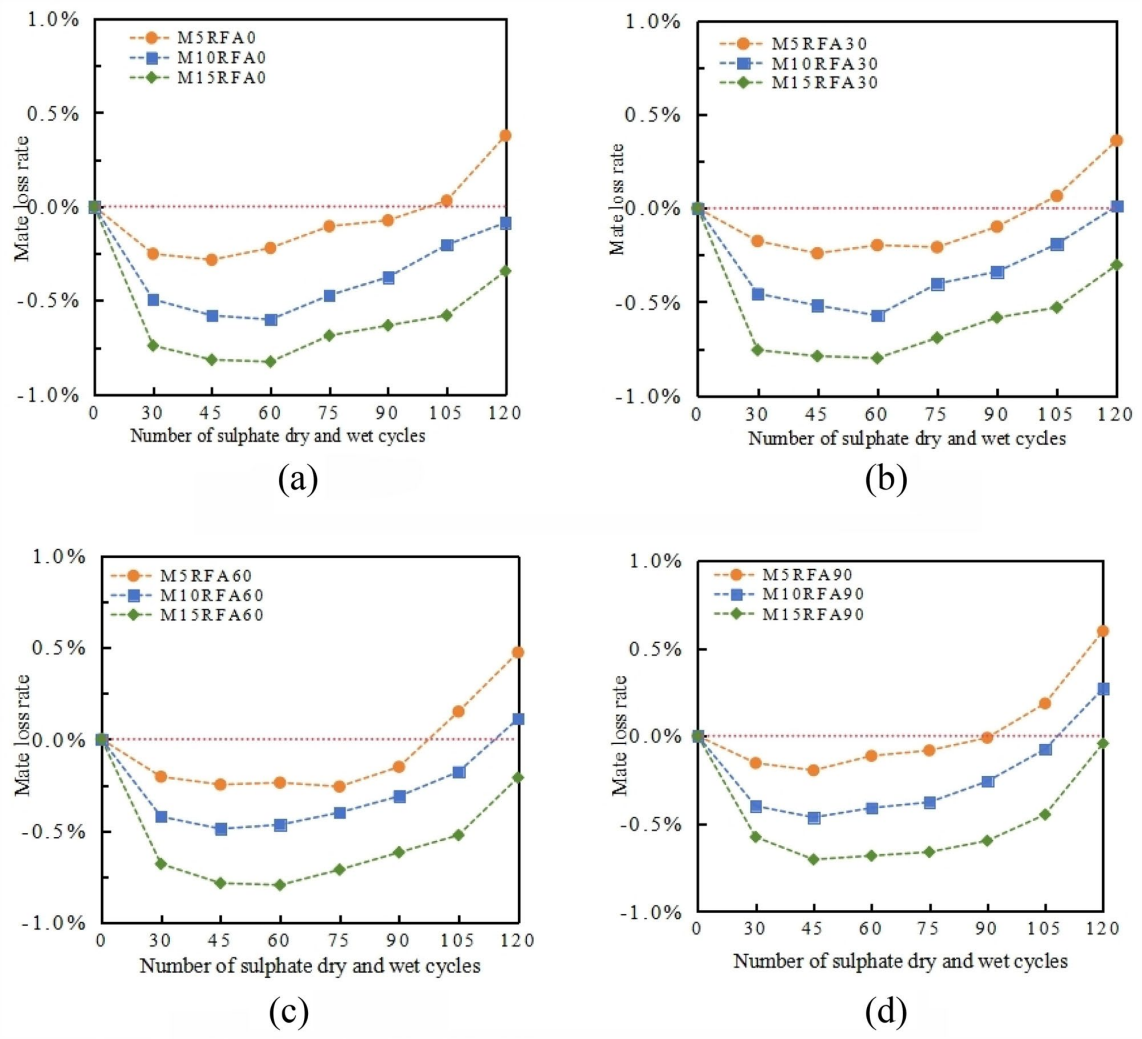
The relationship between the quality loss rate of concrete and the number of cycles under different MK content. (**a**) RFA0, (**b**) RFA30, (**c**) RFA60, (**d**) RFA90.

The mass loss rate of M/RFAC with RFA content of 0%, 30%, 60% and 90% after each sulfate dry–wet cycle varies with the number of cycles, as shown in Fig. 15.

There are significant differences in the mass loss rates of concrete specimens in groups M5, M10, and M15, as the RFA increases, the RFAC mass loss rate also increases, as shown in Fig. 15. When the number of dry–wet cycles is less than 30, the mass increases of M5RFA0, M10RFA0, and M15RFA0 are 0.25%, 0.49%, and 0.74%, respectively. As the erosion continues, the increase in mass after 45 cycles slows down significantly compared to 30 cycles, with increases of only 0.03%, 0.11%, and 0.12% respectively. Within 120 cycles, due to the increase in MK content, the mass loss rate of group RFA0 concrete gradually decreases, with concrete M15RFA0 decreasing by 0.72% compared to M5RFA0. In the RFAC groups RFA30, RFA60, and RFA90, the variation trend of mass loss rate with MK content is similar to that of concrete, the mass loss rate of RFAC increases with the increase in MK content, and there is a large difference in mass loss rate among different MK content levels. After 120 cycles of group RFA0 concrete, concrete with 10% and 15% MK content is still in a state of mass increase, with increases of 0.09% and 0.34% respectively, after 120 cycles of group RFA90 concrete, the mass of M15RFA90 also increases, with an increase of 0.04%. The analysis shows that when MK is mixed with FA and added to RFAC, the harmful pore size is reduced, which improves the internal compactness^28^. This change in internal structure effectively reduces the entry of sodium sulfate solution into the matrix pores, thereby enhancing the resistance of RFAC to sulfate erosion and dry–wet cycle dual effects. According to the experimental results, the optimal MK content for the dual effects of sulfate erosion resistance and dry–wet cycle performance of M/RFAC is 15%.

### Apparent and microscopic analysis

#### Apparent analysis

**Figure 16:**
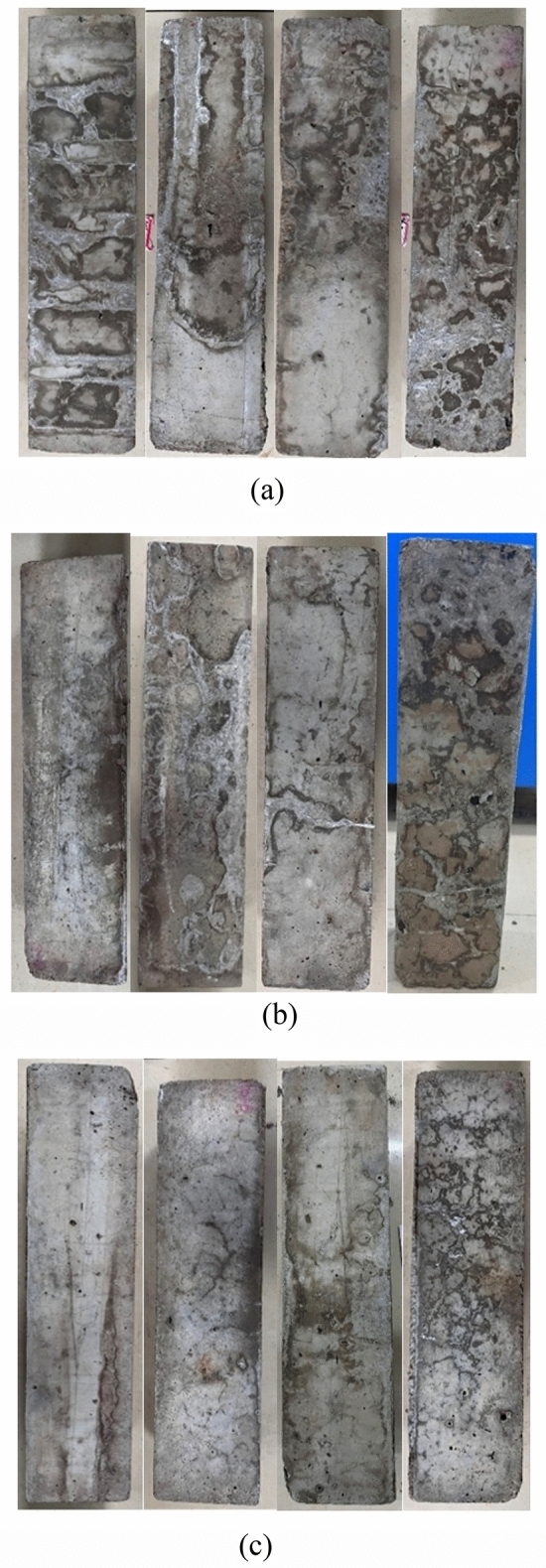
Damage morphology of M/RFAC after 120 erosion cycles. (**a**) Group M5 concrete: M5RFA0, M5RFA30, M5RFA60, M5RFA90, (**b**) Group M10 concrete: M5RFA0, M5RFA30, M5RFA60, M5RFA90, (**c**) Group M15 concrete: M15RFA0, M15RFA30, M15RFA60, M15RFA90.

In the process of sulfate wet-dry cycle alternate corrosion, concrete surfaces exhibit varying degrees of corrosion changes, which are influenced by different amounts of MK, different RFA, and different erosion mechanisms, mainly manifested in surface spalling and pitting. During the wet-dry cycle, the alternation of water evaporation and wetness may cause volume changes and stress concentration in concrete, thereby exacerbating surface spalling and particle detachment; sulfate erosion can cause damage to the concrete’s pore structure, and the wet-dry cycle may further damage the pore structure. The ingress and egress of water can cause expansion and contraction in the pores, increasing pore enlargement and connectivity, enlarging the surface light holes, further weakening the durability and impermeability of the concrete. The appearance damage process of M/RFAC under sulfate-wet-dry cycle dual erosion is relatively slow, with minor differences in appearance damage in the early stage. After 120 erosion cycles, the appearance of each group of M/RFAC is shown in Fig. 16. According to the theory of salt crystallization, salt crystalline damage occurs when the solution concentration reaches saturation^31^. In group a with the lowest MK content, the concrete specimens exhibited a significant presence of white crystals on the surface, along with noticeable grid-like cracks. This indicates that salt crystallization has occurred in the test specimens. Additionally, due to the aggregation of sulfate crystals in the concrete, internal stress is unable to resist the cohesion between aggregate and mortar, leading to damage in corners and edges, partial mortar and aggregate spalling, forming uneven erosion edges, and numerous holes in the surface layer. However, as the MK content increases, the holes relatively decrease. In group c with M15 concrete, only a few of white crystals and cracks appeared. This indicates that with the increase in MK content, the hydration products between aggregate and cementitious materials become denser, the encapsulation of cementitious materials on aggregate becomes more compact, and the connection becomes more reliable.

#### Microscopic analysis

The microstructure of concrete plays a crucial role in the performance and improvement of concrete. The study of concrete microstructure can reveal the internal mechanism and behavior of concrete, and provide powerful theoretical guidance for the study of macroscopic physical and mechanical properties and durability of concrete. Microscopic analysis was conducted on specimens after 120 erosion cycles under M15.

**Figure 17:**
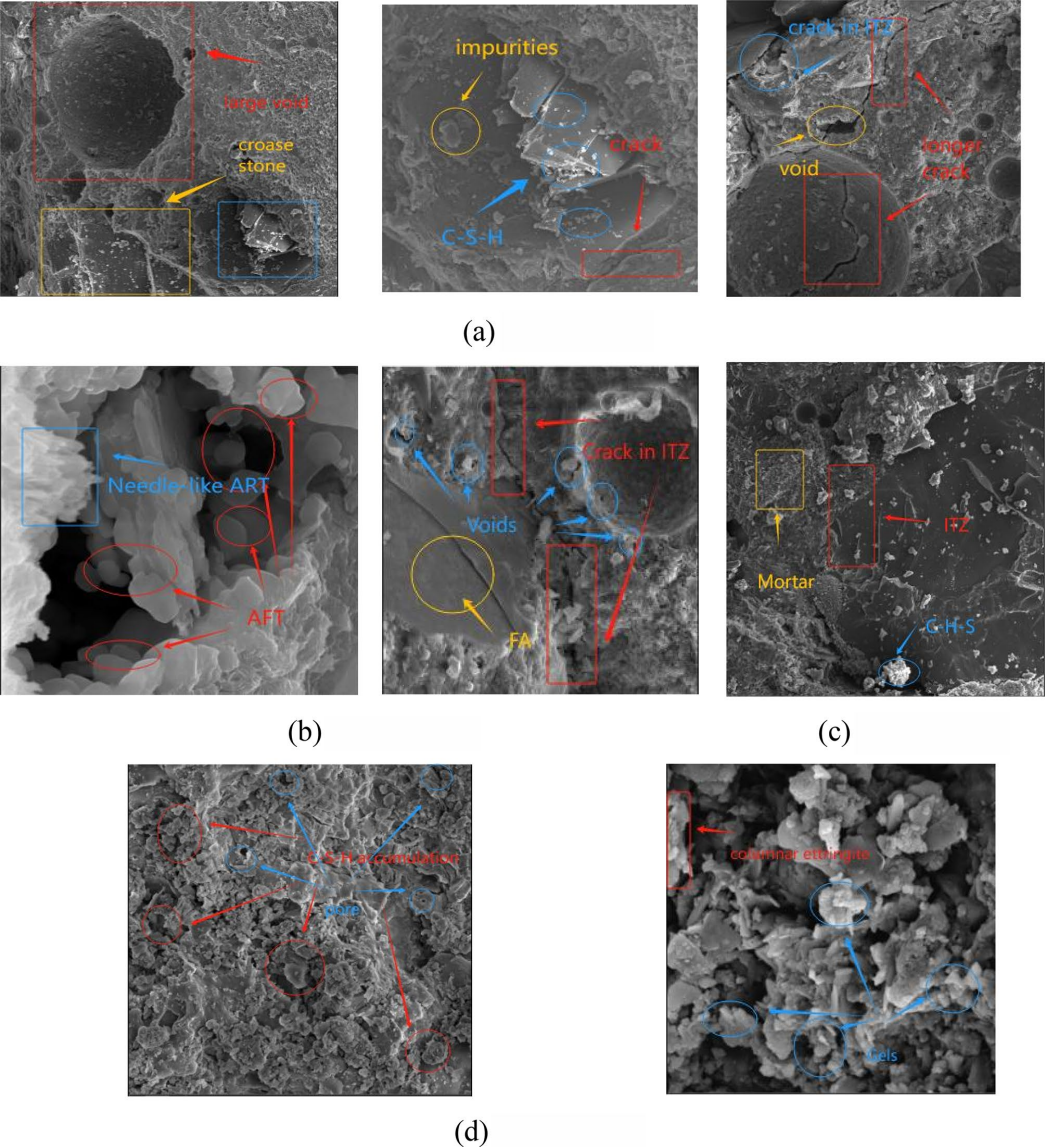
SEM of M/RFA concrete sulfate after 120 dry–wet cycles. (**a**) M15RFA0, (**b**) M15RFA30, (**c**) M15RFA60 and (**d**) M15RFA90.

The scanning electron microscope images (SEM) of the four types of M/RFA concrete after 120 cycles of sulfate wet-dry cycle are shown in Fig. 17. From Figure (a), it can be seen that without the addition of RFA, due to insufficient bonding of hydration products, the micrograph exhibits large pores and cracks, with numerous particle-like protrusions, resulting in uneven hydration products and local formation of a small amount of ettringite^32^. When 30% RFA is added, cracks appear at the ITZ and gradually decrease, and the pores also relatively decrease. This indicates that the small amount of ettringite crystals produced by RFA fills the larger voids and pores, but the small amount of cementitious material produced cannot completely fill the cracks and large voids. With the RFA content increased to 60%, the accumulation of hydration products between aggregate and cementitious materials becomes denser, the quality of ITZ connection is further improved, the number of pores decreases, and the microstructure becomes denser. Because, firstly, after RFA is added, the development of C–H–S gel particles is better, the cementitious particles are stacked^33^ and uniformly distributed on the surface of unhydrated particles, forming a dense microstructure. Secondly, with the increase of RFA, the cementitious particles can effectively fill the voids between cement particles and promote the hydration reaction of cement, refining the matrix pore structure. With further increase of RFA, columnar ettringite appears in the specimens. Under sulfate erosion, the formation and morphology of ettringite cause concrete expansion damage^34^, and the erosion of concrete presents a honeycomb pattern, reducing density and forming a loose porous structure. This finding is consistent with the results of Zhang et al.^35^. Excessive cementitious material cannot be fully compacted, the cracks at the interface between the RFA aggregate and the old mortar are wide, and the volcanic ash in the mixed aggregate MK does not actively participate, leading to more weak points in the internal structure of the matrix, reducing its density. Therefore, it can be concluded that an appropriate amount of RFA can combine with MK and undergo a chemical reaction, effectively preventing the damage of M/RFA and increasing its service life.

### Damage model of recycled concrete under sulphate attack and wet and dry cycles

Based on the evolution process of the material properties of RFAC under the dual action of sodium sulfate erosion and wet-dry cycles, it can be inferred that the" sodium sulfate erosion-wet-dry cycle action" will inevitably lead to the occurrence of durability damage to recycled concrete. Throughout the entire cyclic erosion process, in the early stage of the test, the internal microcracks and micropores of the recycled concrete material are filled with reaction products. In the later stage of the test, due to the large volume expansion and accumulation of reaction products, internal microcracks in the recycled concrete begin to germinate and form, and then expand and connect, thereby causing damage to the internal structure of the recycled concrete. From the above experimental results, it can be seen that the sodium sulfate coupled wet-dry cycle erosion damage process of M/RFAC is an irreversible cumulative damage process, and there are many factors that lead to the deterioration of the durability performance of recycled concrete. This paper mainly considers the generality of the damage evolution calculation model of RFAC under actual sulfate erosion environment, and studies the damage calculation model of M/RFAC, assuming that the initial damage of the damage model is zero, and only considers the influence of RFAC replacement rate and MK amount. As shown in Fig. 13. The relation between the relative dynamic modulus of RFA with the number of cycles under different MK content under the erosion effect, the change rule of the relative dynamic modulus *E*_*(n)*_ of recycled concrete can be roughly divided into three stages: rapid ascent phase a (0–30 cycles), slow descent phase b (30–60 cycles) & rapid descent phase c (60–120 cycles). There is only one inflection point in the damage change process, which conforms to the expression form of a quadratic polynomial. Therefore, the damage model of M/RFAC is selected as the expression form of a quadratic polynomial, that is, the relationship between the relative dynamic modulus of recycled concrete and the number of wet-dry cycles changes as shown in Eq. (7).

7 $$E_{\left( n \right)} = a_{1} n^{2} + a_{2} n + 100,$$

where *E*_*(n)*_ is the relative dynamic modulus value of M/RFAC (%), *n* is the number of wet-dry cycles experienced by recycled concrete, and *a*_*1*_ and *a*_*2*_ are fitting constants.

**Figure 18:**
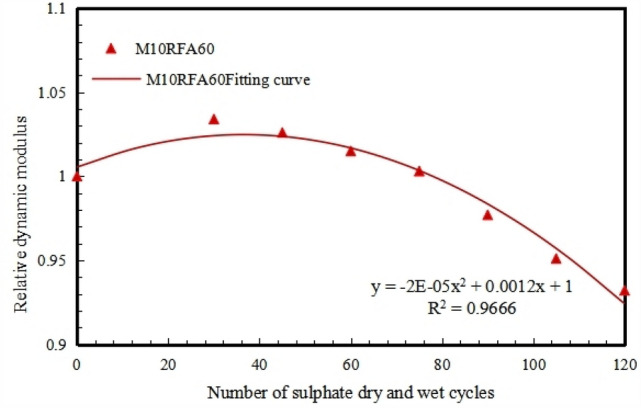
The fitting relationship between the relative dynamic modulus of M10RFA60 and the number of cycles.

Taking the concrete specimen M10RFA60 as an example, the fitting relationship between its dynamic modulus and the number of wet-dry cycles under the dual action of sulfate erosion can be seen in Fig. 18. It can be observed that the fitting curve matches the actual curve well, with a correlation coefficient of 0.97. The fitting relationship is shown in Eq. (8).

8 $$E_{\left( n \right)} = - 2 \times 10^{ - 5} n^{2} + 1.2 \times 10^{ - 3} n + 100.$$

According to the principles of macroscopic damage mechanics of materials^36,37^, the damage variable *D*_*(n)*_ of recycled concrete after n wet-dry cycles can be calculated using the Eq. (9).

9 $$D_{(n)} = 1 - E_{\left( n \right)} ,$$

where *D*_*(n)*_ is the damage value of M/RFAC after different number of wet and dry cycles.

**Figure 19:**
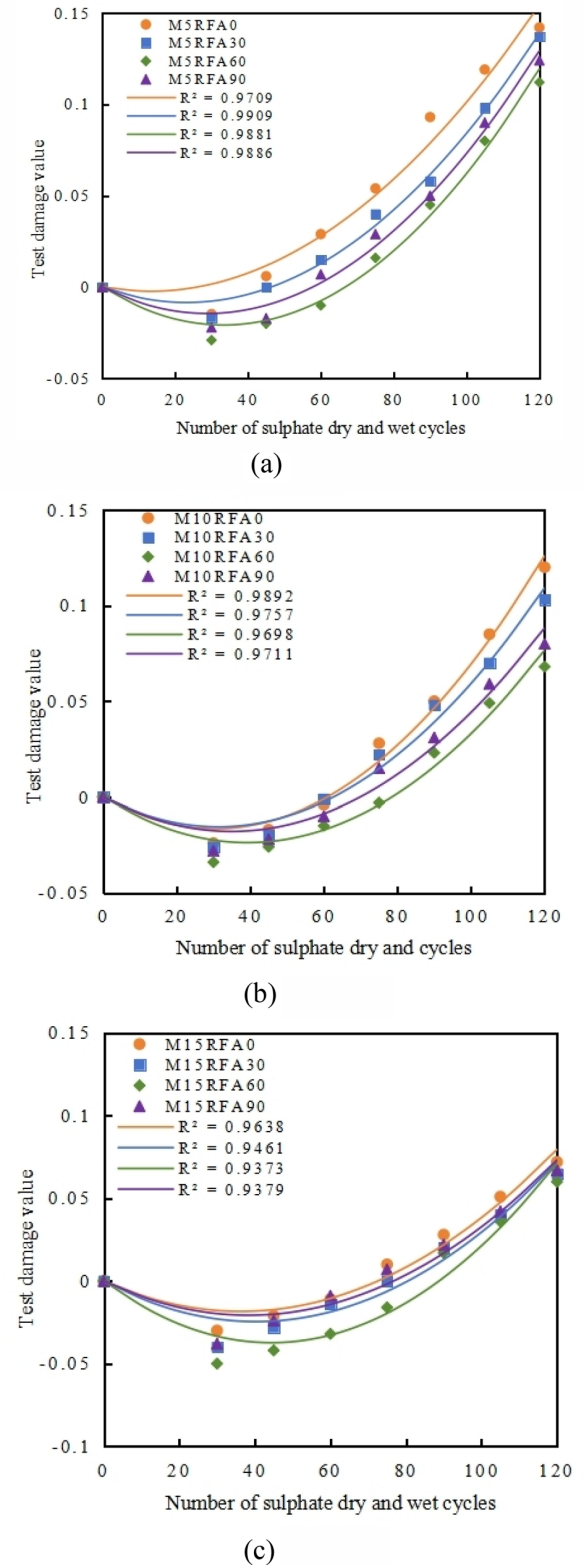
Fitting relationship between test damage and cycle times of concrete with different mix ratio. (**a**) M5, (**b**) M10, (**c**) M15.

Based on the test results in Fig. 18, combined with the Eq. (9), the damage values of concrete after different numbers of wet-dry cycles are calculated. The fitting relationship between the relative dynamic modulus of M5, M10 and M15 group recycled concrete and the number of cycles is obtained from the damage values, as shown in Fig. 19.

It can be observed that the damage of concrete increases with the number of wet-dry cycles, following a basic quadratic polynomial curve. Assuming the damage evolution equation of recycled concrete with the number of wet-dry cycles is as follows: Eq. (10).

10 $$D_{\left( n \right)} = b_{1} n^{2} + b_{2} n + b_{3} ,$$

where *n* is the number of wet and dry cycles of recycled concrete; *b*_*1,*_* b*_*2*_ and *b*_*3*_ are fitting constants.

The damage evolution equation of M10RFA60 recycled concrete with the number of wet-dry cycles is obtained through data fitting. $b_{1} = 2 \times 10^{ - 5}$, $b_{2} = - 1.2 \times 10^{ - 3}$, $b_{3} = 0$_._ The fitting constants *b*_*1,*_* b*_*2*_ and *b*_*3*_ are material parameters of the recycled concrete itself. To further consider the influence of the replacement rate of recycled fine aggregate and the amount of MK in the mix proportion on the coupled damage of recycled concrete, a material correction coefficient *m* for recycled concrete is introduced, as shown in Eq. (11).

11 $$D_{\left( n \right)} = m\left( {b_{1} n^{2} + b_{2} n} \right),$$

where *m* = *m*_*RFA*_* m*_*M*,_ *m*_*RFA*_ and *m*_*M*_ re the coupled damage correction coefficients of recycled concrete specimens under different single factors such as the replacement rate of RFA and the amount of MK. Therefore, the damage evolution equation of recycled concrete under the dual action of sulfate erosion and wet-dry cycles is shown in Eq. (12).

12 $$D_{\left( n \right)} = m_{RFA} m_{M} \left( {b_{1} n^{2} + b_{2} n} \right) = B_{1} n^{2} + B_{2} n.$$

**Figure 20:**
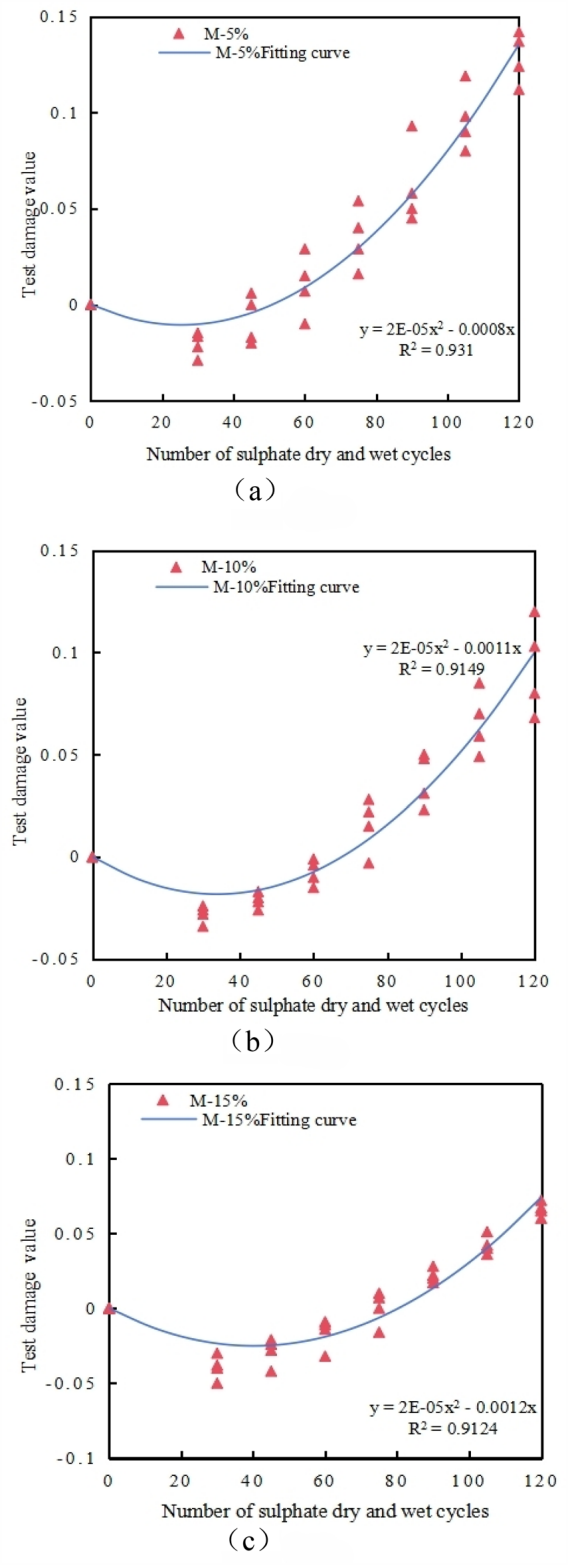
The fitting relationship between the damage of M/RFAC test and the number of cycles under the same MK content and different RFA substitution rate. (**a**) M5, (**b**) M10, (**c**) M15.

From the above Fig. 19, it is found that in this experiment, under the dual action of sulfate erosion and wet-dry cycles, the damage coefficient of concrete is significantly affected by the amount of MK added. Moreover, the damage of recycled concrete *D*_*(n)*_ under the same amount of MK is directly fitted with the number of wet-dry cycles using a quadratic polynomial, as shown in Fig. 20. The damage evolution equation of MK RFAC under the dual action of sulfate erosion and wet-dry cycles can be simplified to Eq. (13).

13 $$D_{\left( n \right)} = B_{1} n^{2} + B_{2} n.$$

The fitting correlation coefficients *R*^*2*^ are all greater than 0.9, indicating that the quadratic polynomial fitting under different MK contents can well express the relationship between concrete damage under the dual action of sulfate erosion and wet-dry cycles in this experiment. Therefore, a damage prediction model for recycled concrete under different MK contents is established, as shown in Table 5.

**Table 5:** Damage prediction model of M/RFAC under sulphate attack and wet-dry cycle.

Content of MK (%)	Damage prediction model
5	*D*_*(n)*_ = 0. 0002*n*^*2*^–0.0008*n*
10	*D*_*(n)*_ = 0. 0002*n*^*2*^–0.0011*n*
15	*D*_*(n)*_ = 0. 0002*n*^*2*^–0.0012*n*

## Life prediction modeling

### Theoretical basis of Weibull distribution

The Weibull distribution function is capable of providing accurate failure analysis and lifetime prediction for small sample data^38^. It is widely used in the durability analysis of concrete materials^39^, which have a minimum safe lifespan compared to the log-normal distribution^40^. In this study, we employ a two-parameter approach to predict the durability life of M/RFA. We assume that the durability life of concrete follows this distribution and estimate the shape parameter and scale parameter to establish the reliability parameters of concrete.

The distribution function *F*_*(µ)*_ is: Eq. (14).

14 $$F_{(\mu )} = 1 - \exp \left[ { - \left( {\frac{\mu }{\lambda }} \right)^{m} } \right].$$

The reliability function *R*_*(µ)*_ is: Eq. (15).

15 $$R_{(\mu )} = 1 - F(\mu ) = \exp \left[ { - \left( {\frac{\mu }{\lambda }} \right)^{m} } \right],$$

where *m* is the shape parameter, *m* > 0;* λ* is the scale parameter, *λ* > 0; *µ* is the time of the sulfate-dry–wet cycle, *µ* ≥ 0.

In addition, the reliability of concrete is inversely proportional to its service time. The reliability of concrete structures decreases with the increase of cycle times. When affected by harsh environmental factors, the decrease in reliability accelerates. Generally, 0 < *R*_*(µ)*_ < 1. If the reliability is less than or equal to 0, it indicates that the concrete structure has failed. The number of sulfate dry–wet cycles that the specimen has experienced when it reaches failure is the predicted life required.

### Parameter estimation using relative dynamic elastic modulus to evaluate parameter Weibull distribution

By evaluating the changes in the relative dynamic modulus of elasticity as the parameter for assessing concrete corrosion degradation damage variables, Eq. (2) can be obtained: Eq. (16).

16 $$D = \frac{{1 - E_{{\text{i}}} }}{0.4} = \frac{{1 - (f_{{\text{i}}}^{2} /f_{{0}}^{2} )}}{0.4} = 1 - \kappa_{2} ,$$

where *E*_*i*_ is the relative dynamic modulus of elasticity. *D* is the degree of damage to the concrete, the normal range is between 0 and 1, in this range indicates that the concrete has not reached the failure state, when* D* ≥ 1, the concrete specimen that is to reach the failure state, when *D* ≤ 0, the concrete is in the stage of reinforcement; 0 < *D* < 1, the concrete is in the normal use of the stage. *f*_*i*_ is the compressive strength after *i* dry–wet cycle. *f*_*0*_ is the initial compressive strength without cycling.

A durability model of M/RFA under sulfate dry–wet cycles is established using the distribution function and reliability function. Since the least squares method is simple and practical^41^, mainly used to calculate the relevant parameters in linear functions, it is used to transform the reliability function into logarithmic form, as shown in Eq. (17).

17 $$\ln [ - \ln R(\mu )] = n\ln \mu - n\ln \lambda .$$

Enable: Eq. (18).

18 $$y = \ln \left[ { - {\text{ln}}R\left( \mu \right)} \right],x = {\text{ln}}\mu ,$$

Simplify to: Eq. (19).

19 $$y = ax + b,$$

Which, $$n = a,\lambda = \exp \left( { - \frac{b}{n}} \right)$$.

The results of parameter calculations for 12 groups of test blocks are shown in Table 6.

**Table 6:** Weibull distributed related parameters.

Specimen number	Shape parameter (n)	Scale parameter ($\lambda$)
M5RFA0	4.306	145.659
M5RFA30	4.447	150.041
M5RFA60	3.363	141.162
M5RFA90	4.962	165.516
M10RFA0	4.373	173.806
M10RFA30	4.599	177.205
M10RFA60	4.014	179.531
M10RFA90	3.842	173.814
M15RFA0	3.376	153.459
M15RFA30	3.101	200.155
M15RFA60	2.766	230.124
M15RFA90	2.854	199.227

### Accelerated life test and reliability analysis of M/RFA based on Weibull distribution

Substitute the values of *m* and *θ* from Table 5 into the above equation to obtain the reliability function *R*_*(µ)*_. Use Origin software to obtain the reliability function *R*_*(µ)*_ curve, as shown in the figure below.

**Figure 21:**
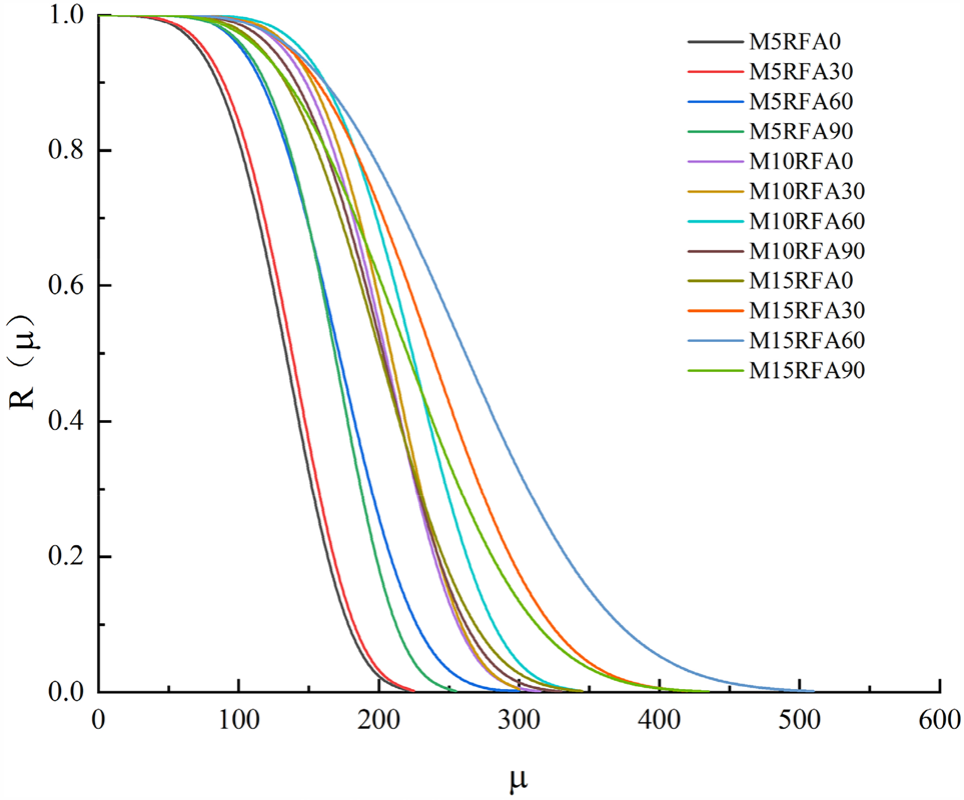
Reliability life prediction curves of different concrete specimens.

Figure 21 illustrates the gradual decrease in reliability of the specimens under sulfate dry–wet cycles. Before 60 cycles, the curve shows a flat stage, indicating that the specimens are undamaged. After 60 cycles, the curve gradually declines, and with more cycles, the decline rate accelerates. Among them, M5RFA0 shows the fastest decline, reaching a reliability close to 0 at 244 and 248 cycles, indicating failure of the specimens. From the graph, it can be observed that with a constant RFA content, the reliability increases with an increase in MK. At M15, the reliability reaches its maximum, with reliability values of 381, 477, 578, and 470 for M15RFA0, M15RFA30, M15RFA60, and M15RFA90, respectively. Under a constant MK content, the reliability of the specimens increases with an increase in RFA content. When the RFA content is 60%, the reliability and basic life of the specimens are maximized, with M5RFA60, M10RFA60, and M15RFA60 reaching a reliability of 0 at 321, 370, and 578 cycles, respectively. However, when the RFA content reaches 90%, the reliability decreases compared to the 60% RFA content. The early stages of the sulfate-dry–wet cycles provide the conditions for secondary hydration of recycled concrete, forming ettringite and calcium silicate hydrate (C–S–H), which fill some of the pores in the concrete, making it denser. With an increase in cycles, the internal stress due to expanding hydration products increases^26^, ending to a decrease in strength. The performance of MK concrete against sulfate dry–wet cycles increases with the level of MK substitution. Firstly, increasing MK reduces the corresponding cement content, reducing the total amount of tricalcium aluminate in the cement paste matrix. Secondly, the particle size of MK is finer than that of cement, resulting in a denser pore structure of MK concrete, thereby enhancing its resistance to sulfate dry–wet cycle erosion^41^. Thirdly, MK has very high pozzolanic activity, and the active Al_2_O_3_ and SiO_2_ in it can react with Ca(OH)_2_ to produce C-S–H gel and hydrated calcium aluminate (C_4_AH_13_, C_3_AH_6_) and hydrated calcium sulphoaluminate (C_2_ASH_8_), improving the pore space^42^ and reducing permeability to refine the ITZ and matrix pores^43^, and the formed ettringite reduces the expansion of concrete. Therefore, the permeability of concrete is reduced, reducing the erosion of concrete by sulfates. Excessive addition of RFA weaken the resistance of the specimens to sulfate. On the one hand, the surfaces of RFA are coated with a large amount of old mortar, leading to excessive reaction of Ca(OH)_2_ with CO_2_ inside the concrete, and also obstructing the bond between the recycled fine aggregate and the cement paste^44^. On the other hand, the high water absorption rate of the recycled fine aggregate causes the specimens to absorb the sulfate solution more rapidly, leading to accelerated damage of the concrete specimens.

## Conclusion

This paper conducted coupled sulfate erosion and dry–wet cycle tests on recycled RFAC with MK. During the dry–wet cycle test, the specimens were subjected to tests for mass loss, cubic compressive strength, and relative dynamic modulus. The following conclusions were drawn from the analysis of the test results, specimen damage morphology, and life damage.

1. With the increase of MK admixture, the compressive strength and relative dynamic elastic modulus of recycled concrete were improved compared with the baseline group, especially the effect of 15% metakaolin admixture was more obvious. In addition, with a certain amount of metakaolin, 60% substitution rate of recycled fine aggregate can effectively reduce the damage of mechanical properties of recycled concrete under the action of dry-wet cycles and sulfate erosion.
2. The cubic compressive strength, relative dynamic elastic modulus evaluation parameter and relative mass evaluation parameter of recycled concrete showed an increasing and then decreasing trend under sulfate dry-wet cycles, and the compressive strength and relative dynamic elastic modulus peaked at M15RFA60.
3. The internal corrosion products in RFAC were observed by SEM, including calcite, gypsum and other crystals. It was found that after 120 times of sulfate dry and wet cycles M15RFA60 the number of internal ettringite increased, a large number of calcite crystals were accumulated intact, and the aggregate was completely encapsulated and formed a continuous structure, which filled the pores to a certain extent and improved the resistance of concrete to sodium sulfate corrosion. The damage model was established by analyzing the experimental data
4. The Weibull function can effectively describe the degradation trend of M/RFAC under sulfate erosion coupled with wet and dry cycles, which can intuitively reflect the specimen's lifetime. According to the reliability function, it can be concluded that MK15RFA has the longest lifetime under sulfate erosion coupled with wet and dry cycles, which is about 578 times.
5. When 15% FA is used as cementing material, it can be combined with 10–15% MK to maximize the content of RFA and auxiliary cementing materials, reduce cement, maximize the content of renewable resources, and improve the environmental friendliness and sustainability of M/RFAC.

This study investigated the effects of a 5% Na_2_SO_4_ solution on recycled concrete after 120 dry–wet cycles, with the best results observed when the RFA content was 60% and the MK content was 15%. Therefore, the authors suggest that by altering the concentration of the Na_2_SO_4_ solution and extending the number of dry–wet cycles, the study can be made more rigorous.

## Data Availability

The data used to support the findings of this study are available from the corresponding authors upon request.
